# The urgency to regulate validation of automated blood pressure measuring devices: a policy statement and call to action from the world hypertension league

**DOI:** 10.1038/s41371-022-00747-0

**Published:** 2022-12-07

**Authors:** James E. Sharman, Pedro Ordunez, Tammy Brady, Gianfranco Parati, George Stergiou, Paul K. Whelton, Raj Padwal, Michael Hecht Olsen, Christian Delles, Aletta E. Schutte, Maciej Tomaszewski, Daniel T. Lackland, Nadia Khan, Richard J. McManus, Ross T. Tsuyuki, Xin-Hua Zhang, Lisa D. Murphy, Andrew E. Moran, Markus P. Schlaich, Norm R. C. Campbell

**Affiliations:** 1 Menzies Institute for Medical Research, University of Tasmania, Hobart, TAS Australia; 2 Lancet Commission on Hypertension Group, London, UK; 3 Department of Non Communicable and Mental Health, Pan American Health Organization, Washington, DC USA; 4 Department of Pediatrics, Division of Nephrology, Johns Hopkins University School of Medicine, Baltimore, MD USA; 5 Istituto Auxologico Italiano, IRCCS, Department of Cardiovascular, Neural and Metabolic Sciences, San Luca Hospital, Milan, Italy; 6 Department of Medicine and Surgery, University of Milano-Bicocca, Milan, Italy; 7 Hypertension Center STRIDE-7, National and Kapodistrian University of Athens, School of Medicine, Third Department of Medicine, Sotiria Hospital, Athens, Greece; 8 Departments of Epidemiology and Medicine, Tulane University Health Sciences Center, New Orleans, LA USA; 9 Department of Medicine, University of Alberta, Edmonton, AB Canada; 10 Department of Internal Medicine, Holbaek Hospital, Holbaek, Denmark; 11 Department for Regional Health Research, University of Southern Denmark, Odense, Denmark; 12 Institute of Cardiovascular and Medical Sciences, University of Glasgow, Glasgow, UK; 13 British and Irish Hypertension Society, Edinburgh, Scotland UK; 14 School of Population Health, University of New South Wales, The George Institute for Global Health, Sydney, NSW Australia; 15 International Society of Hypertension, White Colne, UK; 16 Division of Cardiovascular Sciences, Faculty of Medic2ine, Biology and Health, University of Manchester, Manchester, UK; 17 Manchester Heart Centre and Manchester Academic Health Science Centre, Manchester University NHS Foundation Trust, Manchester, UK; 18 Division of Translational Neurosciences and Population Studies, Department of Neurology, Medical University of South Carolina, Charleston, SC USA; 19 Department of Medicine, Center for Health Evaluation and Outcomes Sciences, University of British Columbia, Vancouver, BC Canada; 20 Nuffield Department Primary Care Health Sciences, University of Oxford, Oxford, UK; 21 Hypertension Canada, Toronto, ON Canada; 22 World Hypertension League, Hong Kong, China; 23 Beijing Hypertension League Institute, Beijing, China; 24 Stroke Foundation, Melbourne, VIC Australia; 25 Resolve to Save Lives, New York, NY USA; 26 High Blood Pressure Research Council of Australia, Melbourne, VIC Australia; 27 Dobney Hypertension Centre, Royal Perth Hospital Research Foundation, Medical School, University of Western Australia, Perth, WA Australia; 28 Department of Medicine, Physiology and Pharmacology and Community Health Sciences, O’Brien Institute for Public Health and Libin Cardiovascular Institute of Alberta, University of Calgary, Calgary, AB Canada

**Keywords:** Disease prevention, Risk factors

This policy statement is intended to be used as a resource for all health professionals and civil society, including regulatory agencies, Ministries of Health and healthcare organizations, to accelerate the availability, affordability, and exclusive use of automated blood pressure measuring devices (BPMDs) that have passed adequate clinical validation testing. In line with guidance from the World Health Organization (WHO) medical device technical series [1], the term clinical validation is the process by which devices are tested for accuracy in healthy people and patients with hypertension, and a clinically validated BPMD is one that has “undergone rigorous, standardized testing against a gold standard [properly calibrated manual auscultatory measurement] to ensure that the device produces accurate measurements” [1] to an internationally accepted standard.

Most automated BPMDs that are marketed for sale globally have not undergone adequate validation testing to ensure clinical accuracy [2–4]. The primary recommendation of this policy statement is for convergence towards the global regulatory requirement for mandatory, independent clinical validation of automated BPMDs according to an agreed universal standard [5, 6]. This will ensure that the accuracy of automated BPMDs is confirmed before being cleared for sale by regulatory authorities, and is an urgent international need advocated by the World Hypertension League, the Lancet Commission on Hypertension and other organizations including the WHO [1, 7, 8].

## Rationale supporting the urgency to regulate validation

High systolic BP contributes to more than 10 million deaths each year and is the single most important modifiable risk factor for cardiovascular disease (CVD) [9]. Controlling hypertension is a global priority to reduce death and disability and subsequent economic costs from CVD [10]. The WHO has advocated for a strategic public health approach to the control of hypertension and developed a series of technical documents, including the HEARTS technical package [11], to assist governments and others engaged in the management of high BP. A foundational aspect to the detection, diagnosis, treatment and control of hypertension is reliable diagnosis which requires an accurate and reproducible method for measuring BP. Critical factors to achieve this include patient preparation, a suitable measurement environment, training and certification of health providers, use of a standardized technique/protocol, and an accurate and precise (preferably automated) BPMD [12].

Automated BPMDs that have been clinically validated for accuracy are widely recommended to be used in favor of manual auscultatory BP measurement methods because the automated component removes observer-related barriers to accurate BP measurement [13, 14]. Automated BPMDs without evidence of having been properly validated for accuracy have greater measurement variability, are less likely to pass static pressure testing, and are more likely to be inaccurate [15–18]. A Canadian study demonstrated that non-validated devices were associated with clinically meaningful discrepancies in measured BP: a > 5 mmHg discrepancy in 69% of patients and >10 mmHg discrepancy in 36% of patients relative to accurately measured BP [19]. Currently, these indadequately validated devices are also commonly used by healthcare providers and by patients for home BP measurement [17, 18], and thus probably contribute to incorrect hypertension diagnosis and management in many individuals. At a population level, even small errors (e.g., 5 mmHg) in systolic or diastolic BP measurement can lead to misclassification of millions of people [2, 12].

The main consequences of inaccurate BP measurement are incorrect diagnosis and deficient medical management, including inappropriate drug treatment. This may manifest as excessive, over-medication for those incorrectly labeled as hypertensive, or lack of or under treatment with therapies proven to reduce CVD events for those incorrectly labeled as normotensive [20]. The above examples of unsafe clinical care lead to increased healthcare risks and also costs that could otherwise be avoided [2, 21–23]. Current estimates indicate that 75–80% of automated BPMDs marketed globally do not have evidence of being adequately clinically validated for accuracy [3, 4]. This is enabled through various regulatory loopholes, and seriously undermines efforts to perform best practice clinical care and efficient CVD prevention [7, 24, 25].

The wide availability of inadequately validated BPMDs also conflicts with essential principles regarding the design and production of medical devices. Specifically, the concept that medical devices must provide accurate measurements, must not compromise an individual’s clinical condition or safety, and must have benefits of use that outweigh any undesirable effects arising from its use [26, 27]. International hypertension societies support the WHO recommendation that adequately validated automated BPMDs must be used in routine clinical management of hypertension [13, 14, 28–31]. There have also been calls to strengthen regulations on the manufacture and marketing of automated BPMDs to address loopholes that allow insufficient proof of accuracy testing [7, 24, 32, 33]. But despite these efforts the global production of inadequately validated automated BPMDs continues to rise in a multibillion-dollar industry, with large annual market growths forecast [34].

Although well-known BP manufacturers support strengthening quality standards for validating automated BPMDs [35], current estimates indicate there could be at least 450 manufacturers producing >3500 unique models, most of which are not validated [3]. Redressing this major international problem requires urgent, consistent and global policy action by government regulatory organizations [36, 37]. The WHO developed a document to guide governments in developing policies that include regulations for automated BPMDs [1]. This document recommends that for routine clinical purposes, including office, ambulatory and home monitoring of BP, that only cuff automated BP devices that have passed accepted international accuracy standards be used (e.g., currently the International Organization for Standardization 81060-2; 2018 protocol) and with validation testing conducted by qualified investigators, independent from the manufacturers.

## Recommended governmental policies

A wide array of government and societal policies are required to ensure the smooth and rapid transition to only allowing the sale of properly validated automated BPMDs. Governments with a strategy for hypertension/CVD/non-communicable disease control will be able to coordinate the development and implementation of needed policies more efficiently. Table 1 provides policy recommendations that governments need to adapt rapidly to ensure only properly validated automated BPMDs are used in routine clinical practice.

**Table 1:** Recommendations for government policies on automated BP measuring devices (BPMDs)^a^.

Build strong regulatory capacity to ensure a smooth and rapid transition to the sole use of validated automated BPMDs for routine clinical use within a strategic approach to hypertension/cardiovascular disease/non-communicable disease prevention and control, emphasizing primary healthcare facilities.
Develop national capacity for independent validation testing of all automated BPMDs, preventing conflicts of interest.
Enable universal access to validation testing protocols and develop checklist/s to provide evidence that validation testing protocols have been followed. This should include a process of regular review and updating.
Regulate the sales (including those online) of automated BPMDs to prohibit the marketing of clinical devices that have either failed validation testing or have not undergone validation testing or where “equivalence” to a validated BPMD is not clearly proven.
Regulate the sale and marketing of automated BPMDs to require packaging that clearly and prominently indicates whether the device has passed validation testing, and for which population (e.g., general population, pregnancy, large arm circumference, atrial fibrillation, children).
Develop an easy-to-access list of validated (including equivalent) automated BPMDs that are readily available in each region. Ensure that this list is regularly reviewed.
Develop policies to ensure equitable and affordable access to validated automated BPMDs including locations where electricity is unreliable.
Develop a procurement policy that includes only validated automated BPMDs for routine clinical use.
Develop technical capacity (e.g., clinical engineers) to appropriately select, maintain and support the use of validated automated BPMDs.

^a^Adapted from ref. [1].

## What can clinicians, civil society, and their organizations do?

Governments may act independently to implement the recent WHO recommendations [1] regarding automated BPMDs, however, the WHO first recommended exclusive use of validated BPMD almost two decades ago [38] and this remains widely unimplemented. Hence, strong advocacy and watchdog action from outside of government is needed to accelerate uptake of the WHO report recommendations [1]. Advocating for and developing a strategic plan to improve hypertension/CVD/non-communicable disease control that includes the rapid transition to sole use of adequately validated automated BPMDs is important.

Adapting this Policy Statement and Call to Action at a national level for use in advocacy is likely to have a larger impact than a global call to action that does not account for the national context. Securing the support of all key national organizations (e.g., stroke and heart foundations, CVD organizations, primary care, important civil society organizations) and forming a health coalition that sustains advocacy actions until government implementation of policies are key to success.

Education of healthcare professionals and the public is also likely to be pivotal. Extensive and global field experience of the authors, as well as recent data [37, 39], indicates that many clinicians are not aware that most automated BPMDs are not properly validated, nor do they recognize the need to routinely use validated automated BPMDs in clinical practice. This deficit in knowledge may undermine advocacy and implementation efforts. Health organizations and civil society can work with accreditation authorities to ensure education and certification throughout training and practice, to provide consistent messaging regarding the need to rapidly transition to the routine use of validated automated BPMDs. All facilities that require accreditation must have adequately validated automated BPMDs available for routine clinical use.

## The way forward

Developing and implementing regulations for the exclusive use of automated validated BPMDs is urgent and a technical imperative. However, it is a complex process requiring political will and coordination of multiple actors at the national and global levels. Civil society and professional and academic organizations play a fundamental role in this context. This process must be a well-planned, progressive and participatory process. Implementation should be gradual to ease acceptance, allow time for realistic replacement of manual BPMDs (and those that are not validated), avoid high costs, and avoid challenges for manufacturers and distributors [39].

For instance, in the region of the Americas, 22 countries led by the Ministries of Health, in collaboration with local stakeholders and the Pan American Health Organization (PAHO), have initiated a set of actions to improve the regulatory landscape and update the procurement mechanisms to promote the exclusive use of validated automated BPMDs [36] with an emphasis in primary healthcare, where most of the persons with hypertension and other non-communicable diseases are managed. These actions have been coordinated through the HEARTS in the Americas program, which is a comprehensive risk reduction model of hypertension and CVD risk management implemented across the region [40, 41]. An important first step was to understand the regulatory frameworks governing the accuracy of automated BPMDs across different countries. These frameworks were found to be weak, fragmented, and lacking both policies and regulations to promote the exclusive use of validated automated BPMDs [32].

Other steps involved creating awareness through technical meetings with regulatory authorities and Ministries of Health to explore actions to strengthen regulations and create resources to assist policy makers, health professionals, regulatory agencies, professional societies, and the public [42]. Training workshops were provided on conducting validation studies to build national capacity in specific countries and practical guides have been published [43]. Information on finding validated automated BPMDs was published [44] and technical resources were listed on the PAHO website [45, 46]. PAHO also published a guidance document to contribute to meeting these recommendations by providing a practical tool for governments to improve their national regulatory frameworks to improve accuracy of automated BPMDs, in turn contributing to the exclusive use of validated automated BPMDs in primary healthcare facilities by 2025 [40].

In conclusion, accelerating the uptake of adequately validated automated BPMDs for routine clinical use is important in the global and national strategy to enhance hypertension/CVD/non-communicable disease control. This effort is consistent with WHO recommendations [1] as well as the global commitment to remove all mercury containing medical devices, including sphygmomanometers, because of its environmental hazard. Equally compelling is the extensive data indicating that manual auscultatory aneroid BP devices are often out of calibration, lack maintenance, and are rarely tested for calibration in clinical practice [47, 48]. The main weakness of automated BPMDs is the lack of regulatory requirements to validate devices for accuracy and precision before receiving regulatory clearance to market and sell. This policy statement is intended to be used, but not limited, by national health and civil society members and organizations in advocacy—and watchdog—to support governments developing and implementing policies, including regulations to accelerate the routine use of appropriately validated automated BPMDs in clinical practice.

## Disclaimer

PO is a staff member of the Pan American Health Organization. HEARTS in the Americas is an initiative of the Pan American Health Organization. However, the authors alone are responsible for the views expressed in this article; those views do not necessarily represent those of the Pan American Health Organization.
